# P-1793. Consensus Guidelines for the Clinical Validation and Interpretation of Respiratory Metagenomic Data from the UK National Health Service Respiratory Metagenomics Network

**DOI:** 10.1093/ofid/ofaf695.1962

**Published:** 2026-01-11

**Authors:** Adela Alcolea-Medina, Robbie Hammond, Catherine Houlihan, Ana Soares, Kordo Saeed, Chloe Myers, Jamie Whitehorn, Rahul Batra, Theodore Gouliouris, Judith Breuer, Julianne Brown, Gaia Nebbia, Jonathan Edgeworth, Luke Blagdon Snell

**Affiliations:** KCL, London, England, United Kingdom; UCL, London, England, United Kingdom; UCL, London, England, United Kingdom; UCL, London, England, United Kingdom; University of Southampton, Southampton, England, United Kingdom; Cambridge University Hospitals NHS Foundation Trust, Cambridge, England, United Kingdom; Guy's and St. Thomas' NHS Foundation Trust, London, England, United Kingdom; NHS, London, England, United Kingdom; University of Cambridge, Cambridge, England, United Kingdom; UCL, London, England, United Kingdom; Great Ormond Street Hospital, London, England, United Kingdom; KCL, London, England, United Kingdom; King's College London, London, England, United Kingdom; KCL, London, England, United Kingdom

## Abstract

**Background:**

Metagenomic sequencing is increasingly used in respiratory diagnostics, yet consistent standards for clinical validation and interpretation are lacking. The UK National Health Service Respiratory Metagenomics Network convened a multidisciplinary group to formulate consensus guidance.

**Methods:**

A targeted literature review, expert-panel discussions and iterative refinement of guidance on pathogen significance and reporting were conducted. Two national consultation rounds and one Delphi round was used to define consensus. Statements achieving greater than 80% agreement advanced to guidance. Areas in which the evidence base was limited or consensus could not be reached have been flagged as priorities for future investigation and further guideline refinement.

**Results:**

Supporting evidence is given for consensus guidance. Guidance for reporting included consideration of specimen type, relative abundance and clinical context. Mandatory escalation for detections of alert organisms were agreed, such as Group A Streptococcus and *Pneumocystis jirovecii.* Suppression criteria for organisms of doubtful significance such as commensal oral flora, anaerobes and *Tropheryma whipplei* when clinical evidence is absent are given. Interpretative comments for herpesviruses were advised. Future work should correlate i) bacterial read counts with semi-quantitative growth in culture and ii) cycle threshold value with significance for viruses and atypical pathogens.

**Conclusion:**

These consensus guidelines provide the first framework for validating and reporting respiratory metagenomic findings across laboratories.

**Disclosures:**

Adela Alcolea-Medina, PhD, Synnovis: AAM is inventor of a patent used for human DNA depletion in metagenomic assays Rahul Batra, MD, Guy's and St. Thomas' NHS Foundation Trust: RB is inventor of a patent used for human DNA depletion in metagenomic assays Jonathan Edgeworth, MB BChir, Oxford Nanopore Technologies: Part time employee|Oxford Nanopore Technologies: Stocks/Bonds (Public Company)

